# Birth Weight and Early Weight Changes in Newborn Dogs: Analyzing Key Influencing Factors in Dobermann Puppies

**DOI:** 10.3390/ani16091410

**Published:** 2026-05-05

**Authors:** Jasmine Fusi, Roberta Bucci, Claudia Scabrosetti, Massimo Faustini, Maria Cristina Veronesi

**Affiliations:** 1 Department of Veterinary Medicine and Animal Sciences, Università Degli Studi di Milano, Via dell’Università 6, 26900 Lodi, Italy; massimo.faustini@unimi.it (M.F.); maria.veronesi@unimi.it (M.C.V.); 2 Department of Clinical Sciences and Translational Medicine, University Degli Studi Roma “Tor Vergata”, Via Montpellier 1, 00133 Roma, Italy; rbuccivet@gmail.com; 3 Ambulatorio Veterinario Claudia Scabrosetti, Via Mazzini 1, 27041 Casanova Lonati, Italy; ambvet.scabrosetti@gmail.com

**Keywords:** newborn dog, birthweight, early growth, growth chart, influencing factors

## Abstract

Birthweight (BW) and early weight changes are important prognostic factors in dog neonatology, but are influenced by the breed. The present study aimed to evaluate BW and early growth in a single breed, Dobermann, and single breeding facility for a better evaluation of data and analysis of influencing factors. In 364 puppies born by elective cesarean section, the ROC curve and cut-off weight values associated with the risk for death within 15 days after birth were defined for BW and Day 1 weight (D1W). Both BW and D1W were influenced by newborn’s sex (*p* < 0.05), maternal age (*p* < 0.05) and maternal bodyweight (*p* < 0.05), litter size (*p* < 0.05), and “litter effect” (*p* < 0.05). The weight charts for the first 15 days after birth were constructed for female and male puppies, and weight percentage variation analysis of the first 5 days after birth showed that the nadir of weight loss occurred around 1 day after birth, followed by BW recovery within 3 days, and doubling BW around 12–14 days. Therefore, BW (*p* < 0.001), D1W (*p* < 0.001) and early weight changes are important factors for prognosis and growth monitoring, allowing the prompt detection of those puppies needing special care. However, the influences of sex-related differences and maternal factors must be considered.

## Simple Summary

In dog neonatology, birthweight (BW) and early weight changes are reliable and good indicators of early survival prognosis. However, both factors are strongly influenced by the marked differences among canine breeds. Studies focusing on single breeds are, therefore, important for a better definition of reference ranges, patterns of growth, and the correct analysis of influencing factors. The present study, performed on a single breed in a single breeding facility, removed the confounding effect of breed and mitigated the effect of different management. The results obtained from 364 puppies from 38 bitches submitted to elective cesarean section confirmed the role of BW and weight on Day 1 (D1W) as prognostic factors in relation to survival at 15 days after birth. A significant influence of maternal age and bodyweight, litter size, “litter effect”, and sex of the newborn on BW and D1W was found. Weight charts were constructed for females and males, and weight percentage variation in the first 5 days after birth showed the time of weight nadir, for BW recovery, and for BW doubling, slightly different between females and males. Therefore, this study confirmed that BW and early weight changes are important for monitoring early growth in puppies, but breed-specific data are necessary.

## 1. Introduction

The neonatal mortality rate in puppies is reported to range on average between 10 and 15% [1,2,3], with most deaths occurring during the first days after birth (2 to 8 days) [1,4,5,6].

The availability of prognostic factors for puppy survival represents, therefore, an important tool in canine neonatology. Among them, birthweight, early neonatal weight loss, or insufficient weight gain have been reported as feasible and easy-to-collect prognostics for newborn outcome parameters [3].

Birthweight (BW) is the reflection of the just completed intrauterine life and intrauterine development, determining the ability of the newborn to cope with the extrauterine life [2,7]. Many studies have evidenced the relation between low BW and neonatal mortality [3,5,8,9,10], and that the BW is strongly influenced by different canine breeds [3,9,10,11,12]. Moreover, BW was reported to be different between the two sexes by some authors [11,13,14,15,16], even if others did not find sex-related differences [10,13]. Maternal factors, such as parity and bodyweight, were also considered as possible BW-influencing factors. According to parity, heavier puppies were born by lower parity bitches in a study on Labrador dogs [14]. Groppetti et al. [9] found an influence of maternal age on BW, with lighter puppies born by bitches aged less than 2 and more than 8 years. The maternal weight can also influence BW, even if this finding is often drawn from studies involving multiple different-sized canine breeds [9,10,11], characterized by different bodyweights. However, some studies on single breeds [14,15] have also found a relation between increasing maternal bodyweight and heavier puppies. The litter size was also reported to be inversely related to BW [10,11,12,14] and is influenced by the breed [9,17]. Recently, some authors [17] proposed the evaluation of the ratio between neonatal bodyweight and maternal bodyweight expressed as a percentage in different breed body size categories to promptly identify puppies requiring additional care.

Besides BW, early weight changes were also considered important prognostic factors for newborn puppies [3,5]. Although some authors did not find a weight loss within 48 h after birth in three canine breeds [12], puppies can physiologically lose not more than 4–5% of BW during the first 24–48 h after birth [13], partly due to meconium and urine loss. After this time, BW recovery should occur within 3–5 days after birth [13], and is followed by a subsequent weight gain, doubling BW during the second week after birth. However, the time for doubling BW was reported to be influenced by the breed [13]. Unsatisfactory weight gain in the first 2 days was found to be a predictor of mortality [5]. For these reasons, focused attention on the puppy’s weight changes during the first 5 days after birth is very important for quickly detecting those newborns with unsatisfactory growth and requiring appropriate management [18].

However, the studies of the last two decades have evidenced that BW, early weight changes, and most of the influencing factors are breed-dependent, so that breed-specific data are needed for the correct monitoring of puppies at birth and during the neonatal period [3,4,5,9,10,11,19,20]. Because the hundreds of canine breeds prevent the availability of data specific to each breed, many studies have provided data stratified according to breed body size, not considering the morphological peculiarities of each breed, even within the same size category [5,6,17,21,22].

Thus, despite the great number of studies reporting data about BW and neonatal weight changes in dogs, most of the information was obtained from studies involving multiple breeds, in which the pattern of weight changes and the impact of possible influencing factors have been confounded by the various breeds included.

Therefore, some aspects of BW and early neonatal weight changes still deserve to be better elucidated. To this purpose, the present study aimed to investigate BW and early weight changes in a single canine breed, with dogs all belonging to a single breeding facility, avoiding the possible influence of the breed and mitigating the effect of different managements. In detail, this study aimed to (1) detect maternal (maternal age, parity, bodyweight, litter size, and “litter effect”, which means the possible effect played by different mothers on BW, as reported by [5]) and neonatal factors related to puppy survival 15 days after birth; (2) define the best BW cut-off value for the risk of death within 15 days after birth and assess the possible influence of maternal factors (maternal age, parity, bodyweight, litter size, and “litter effect”) and neonatal sex on BW; (3) detect the time in which the nadir of weight will occur and the best cut-off for the risk of death within 15 days after birth, and assess the possible influence of maternal factors (maternal age, parity, bodyweight, litter size, and “litter effect”) and neonatal sex on the weight at nadir; (4) provide the weight charts for all born alive puppies, and separately for females and males; and (5) focus on weight percentage variation in the first 5 days after birth in comparison with BW and assess the possible influence of maternal factors (maternal age, parity, bodyweight, litter size, and “litter effect”) and neonatal sex.

## 2. Materials and Methods

### 2.1. Ethics

This study was conducted in accordance with EU Directive 26 June 2019 and approved by the Università degli Studi di Milano Ethical Committee (OPBA) with protocol OPBA_33_2021. Written informed consent was signed by the owners, giving permission to submit each female dog to an elective C-section and allowing the collection of data for research purposes.

### 2.2. Animals, Study Design, and Inclusion Criteria

This study was performed on Dobermann dogs belonging to a single breeder and housed in a single breeding facility. Only female dogs found to be healthy by a clinical examination (sensorium assessment, checking for mucous membrane color, capillary refill time, dental health, lymph nodes, pulmonary and cardiac auscultation, abdominal palpation, rectal temperature, femoral pulse, respiratory pattern, coat, any abnormal behavior or alteration in movements or gait) and submitted to the common vaccination and parasite prophylaxes, with BCS 5/9 [23] at mating, fed with commercial food (Royal Canine^®^, Amargues, France: Adult Maxi and later, during pregnancy, Maxi Starter), and housed in a single kennel with indoor and outdoor spaces, were enrolled. From the onset of proestrus, all the female dogs were monitored through serial vaginal smears every 48–72 h and through the assay of plasma progesterone concentrations performed every 48 h from the beginning of the signs of cytological estrus. Based on these parameters, all the female dogs were submitted to a single mating, performed with a male of proven fertility, 48 h after the estimated ovulation, when progesterone plasma concentrations ranged between 4 and 10 ng/mL [24]. Only bitches with a history of previous and current normal pregnancies, post-partum and lactations, were enrolled [25]. In all of them, an elective cesarean section was planned at term, because of the risk for dystocia due to large litter sizes or individual predisposition to whelping troubles [26], on the basis of multiple parameters, as reported by [27,28]. At 25–28 days after the estimated ovulation, pregnancy was checked by ultrasonographic examination, and the pregnant female dogs were submitted to measurement of the inner chorionic cavity (ICC) for the first calculation of the parturition date and for the evaluation of fetal viability by assessment of early fetal cardiac motion. At 40–45 days after ovulation, a second ultrasonographic evaluation was performed to assess the normal course of pregnancy and the correct development and well-being of the fetuses, and to measure the biparietal (BP) diameter for an additional calculation of the parturition date. The day for performing elective cesarean section was scheduled on the basis of the date of ovulation, based on the measurement of plasma progesterone concentrations, ICC and BP. However, in the last 5–3 days before the estimated date of parturition, bitches were submitted to a clinical and ultrasonographic monitoring of the mothers and fetuses well-being. Fetal maturity was estimated by assessing gastrointestinal motility detection [29]. On the estimated day of parturition, bitches were submitted to elective cesarean section only if plasma progesterone concentrations were ≤2 ng/mL [27]. The same anesthetic and surgical protocols, aimed at minimizing the possible negative effects on newborn viability and mothers’ well-being, were performed, as previously reported [27]. Briefly, atropine (0.02 mg/kg IM) and metoclopramide (0.2 mg/kg SC) were used for premedication, cefazolin was injected (25 mg/kg IV), an oxygen mask was applied, and 15 min later, induction was performed with propofol (4–6 mg/kg IV). Anesthesia was then maintained with isoflurane and oxygen. Lidocaine (2 mg/kg) was splashed on the surgical incision before the subcutaneous layer was closed. Immediately after the last fetus extraction, tramadol (3 mg/kg IV) and oxytocin (0.15 IU/kg IM) were administered to the bitches. All the surgeries were performed by the same surgeon, and two to three expert neonatologists took care of the newborns as soon as they were extracted from the uterus, providing professional assistance. In all newborns, vitality was assessed by the Apgar score at 5 min after birth [28]. All puppies were scored 6–10. At that time, a first stabilization was already performed (drying with soft clothes, clearing of the airways, tactile stimulation by thorax rubbing, and umbilical cord clamping). After these first cares (provided to all the newborns within one minute after birth), and after checking for the absence of malformations, additional assistance was provided to each newborn, depending on their class of vitality. In some cases, a small nasal enema was used to remove fluids from the oral cavity, and when needed, a flow-by oxygen administration was provided. No puppies required intensive resuscitation. After this assistance, they were put in an incubator with controlled temperature [30].

All born alive puppies were immediately identified by different colored collars. Puppies born with gross physical malformations or stillborn were excluded. For each parturient bitch, the following data were recorded: age, parity, bodyweight before pregnancy (pre-pregnancy bodyweight), litter size, considering the total number of born puppies per litter, and distinguishing between stillborn, born alive but dead within 15 days after birth, and born alive and surviving at 15 days after birth, and the sex of each newborn. Birthweight was recorded with a digital scale (PetScale, SecurLab^®^, Rome, Italy) calibrated on 1 g as soon as the birth assistance was ended, before the first sucking, and then the individual’s weight was measured daily, always at the same time of the day, always by the same person. A daily follow-up to update the general conditions of mothers and puppies was provided by the breeder for the following 15 days after birth. The ratio between the mean BW and mean maternal body weight expressed in percentages was recorded for puppies alive at 15 days after birth and for puppies dead within 15 days after birth [17]. All puppies were exclusively bitch fed, and no issues were detected during the bitch feeding period.

### 2.3. Statistical Analysis

The logistic regression analysis was used to assess the possible influence of maternal age, parity, body weight, litter size, “litter effect”, newborn sex, and newborn BW on survival up to 15 days of age. For a second time, a receiver operating characteristic (ROC) curve was built, and the Youden test was used to detect the best cut-off value for BW and weight on Day 1 in relation to newborns’ survival 15 days after birth. Subsequently, a linear regression analysis was performed to assess the potential influence of maternal age, parity, body weight, litter size, “litter effect”, and newborn’s sex on BW and weight on Day 1 after birth. A polynomial degree-5 regression model was applied to assess variance in weight over the time of study and to create 3 curves of growth: 1 for all born alive puppies, and the other 2 separated by sex. Finally, an ANCOVA was used to assess the weight percentage changes, relative to BW, during the first 5 days after birth, in male and female born-alive puppies. Day and sex were considered fixed factors, while maternal age, parity, maternal weight, litter size and “litter effect” were considered covariates. Significance was set at *p* < 0.05 (JMP version 19.0; Jamovi Cloud 2.6.44).

## 3. Results

### 3.1. Clinical Data

From the 38 bitches, a total of 372 puppies were born, five stillborn (1.4%), three (0.82%) who were affected by severe malformations and underwent compassionate euthanasia immediately after birth, and 364 (97.8%) who were born alive and normal. Twenty-five puppies, 12 females and 13 males, died within the first 15 days after birth (6.7%) (Table 1), while 339/372 (91.1%) were alive 15 days after birth.

The five stillborn puppies, two females and three males, excluded by the analysis of weight changes in the following 15 days, showed a BW of 432 ± 43.24 g.

The data, expressed as means ± SDs and (min–max) of maternal age, parity, pre-pregnancy bodyweight, litter size, and the male/female ratio of the 38 studied bitches, are reported in Table 2.

The ratio between the mean BW and mean maternal body weight expressed in percentage in puppies alive 15 days after birth was 1.3%, while that of puppies that died within 15 days after birth was 1%.

### 3.2. Birthweight

**Figure 1:**
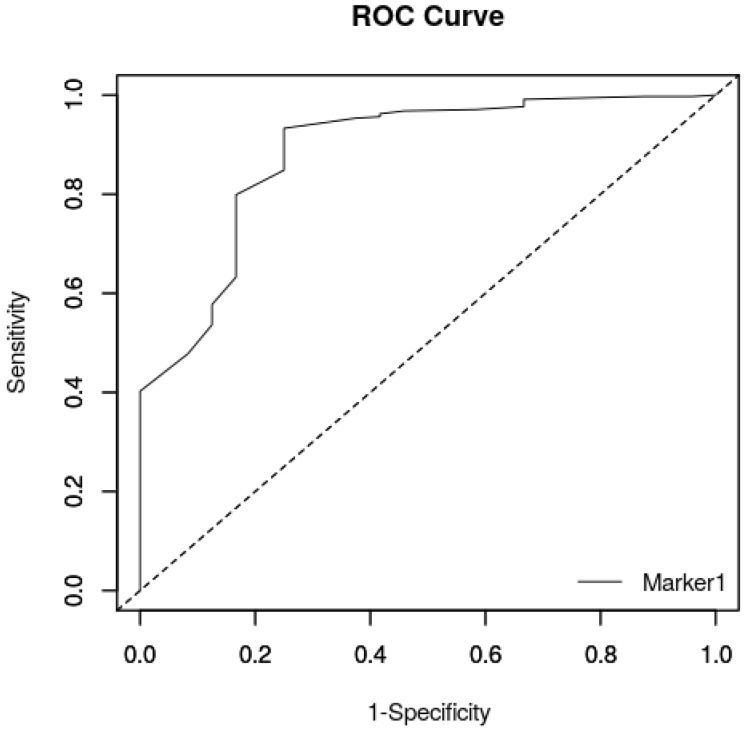
Area under curve related to BW obtained from the 364 born-alive puppies.

The logistic regression showed that BW was associated with survival until 15 days after birth (*p* < 0.001). Therefore, a ROC curve was built, and the Youden test was used to detect the best BW cut-off value for survival to 15 days after birth. The area under the curve was statistically significant: AUC = 88.4% (95% CI = 80.8–95.9) (*p* < 0.001) (Figure 1).

The Youden test showed that the best BW cut-off value to predict the outcome 15 days after birth was 340 g (Table 3).

The statistical analysis showed a significant (*p* < 0.05) difference in BW between males and females, with higher BW in males, 438.8 g (95% CI: 421.1–456.5 g), than in females, g (95% CI: 394.9–438.1 g).

Furthermore, the linear regression showed that BW was also significantly influenced by maternal age (*p* < 0.05), with heavier puppies born by younger mothers; by pre-pregnancy maternal body weight (*p* < 0.05), with heavier bitches giving birth to heavier puppies; by litter size (*p* < 0.05), with heavier puppies belonging to smaller litters; and also by the “litter effect” (*p* < 0.05).

### 3.3. Weight Changes Within 15 Days After Birth

**Figure 2:**
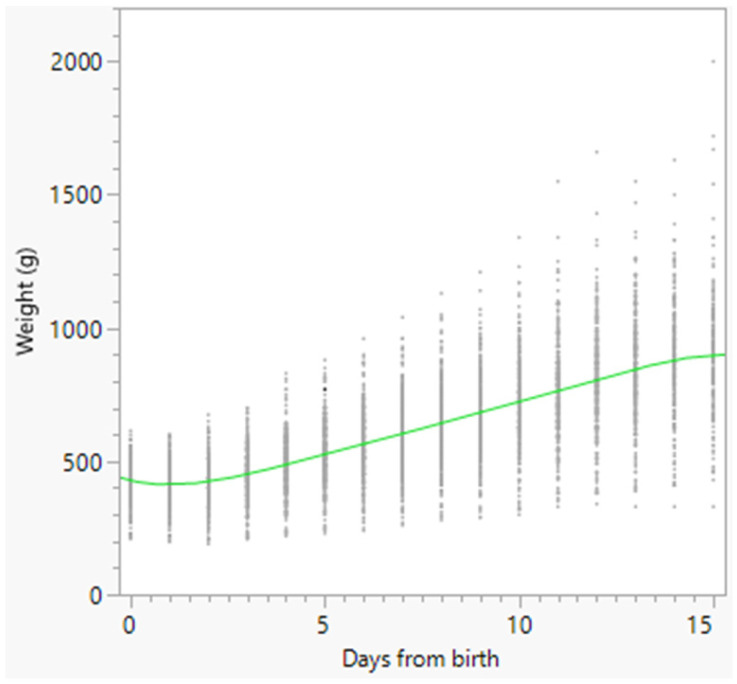
Polynomial degree-5 regression model growth chart of the 364 born-alive puppies. Dots represent single weight measurements; the green line represents the polynomial degree-5 regression.

**Figure 3:**
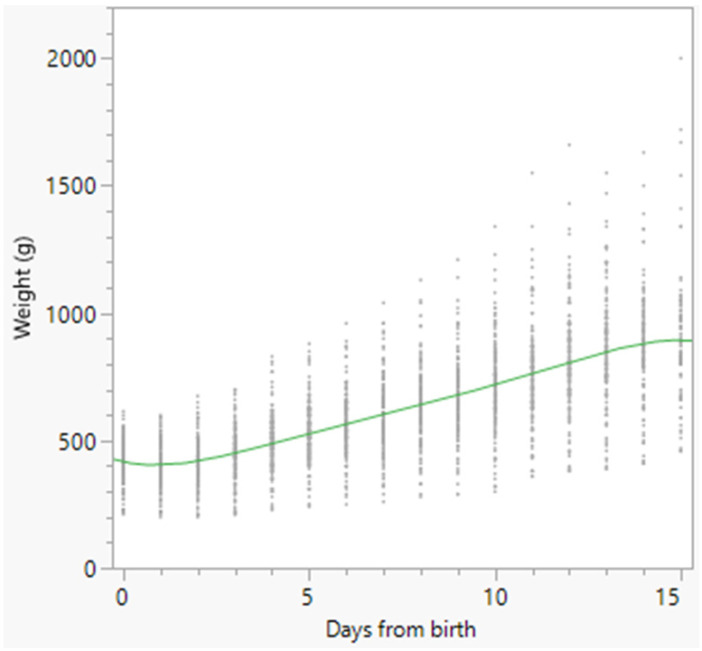
Polynomial degree-5 regression model growth chart of the 175 female born-alive puppies. Dots represent single weight measurements; the green line represents the polynomial degree-5 regression.

**Figure 4:**
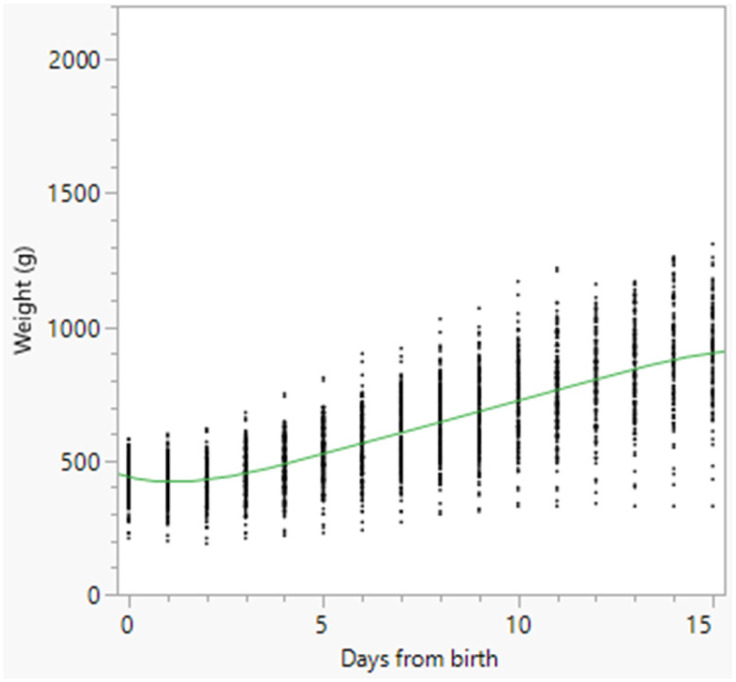
Polynomial degree-5 regression model growth chart of the 189 male born-alive puppies. Dots represent single weight measurements; the green line represents the polynomial degree-5 regression.

Growth charts of the total number of born alive puppies, and of females and males separately, were constructed using a polynomial curve and are reported in Figure 2, Figure 3 and Figure 4, respectively.

For a better understanding of weight changes during the first 15 days after birth, the mean changes in weight and % of variation every 3 days, in comparison with BW (100%), in the total number of born alive puppies, and in females and males, are reported in Table 4.

**Figure 5:**
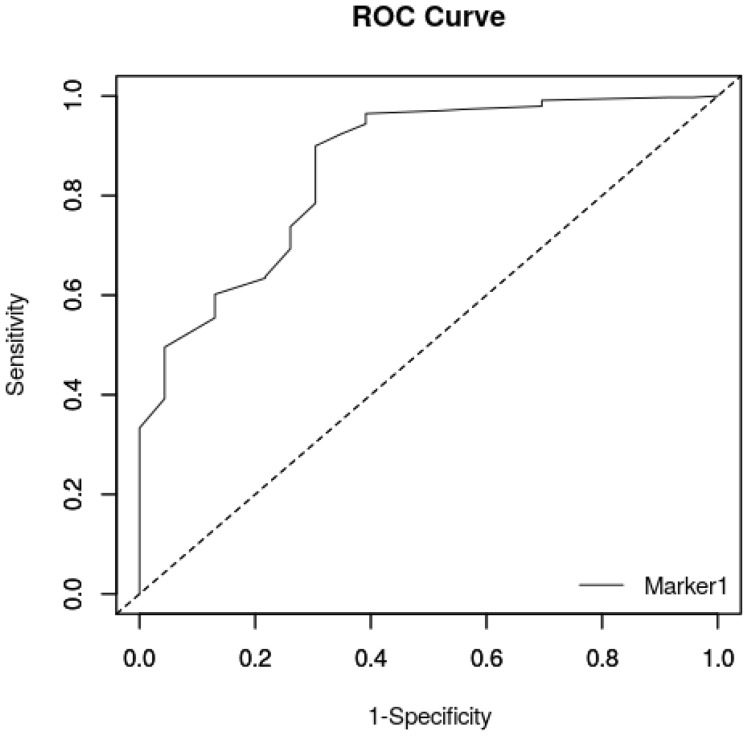
Area under curve related to D1W obtained from the 364 born-alive puppies.

The nadir of weight was observed in all puppies on Day 1, on Day 0.87 in females, and on Day 1.15 in males (Figure 2, Figure 3 and Figure 4). Puppy’s body weight on Day 1 after birth (D1W) was also significantly associated with survival at 15 days after birth (*p* < 0.001), and a ROC curve (Figure 5) was built, followed by the Youden test, detecting the best D1W cut-off value at 340 g (Table 5). The area under the curve was statistically significant: AUC = 85.6% (95% CI = 77.3–93.9) (*p* < 0.01).

The statistical analysis also showed that D1W was influenced by maternal age (*p* < 0.05), with heavier puppies belonging to older mothers; by pre-pregnancy maternal bodyweight (*p* < 0.05), with heavier bitches related to heavier puppies; by litter size (*p* < 0.05), with heavier puppies belonging to smaller litters; and by “litter effect” (*p* < 0.05).

The mean time for doubling BW was 13.3 days in all puppies, 12.6 days in females, and 14.0 days in males.

A focus on weight percentage variation in comparison with BW during the first 5 days after birth showed a significant effect of the Day and of the sex of the newborn. Weight percentage variation was significantly different among all days (*p* < 0.05), except between Day 1 and Day 2. Data regarding differences between female and male puppies alive 15 days after birth, in weight percentage variation during the first 5 days after birth, are reported in Table 6.

The results showed that, in females, the percentage of weight loss, lower than that observed in males, occurred only on Day 1, while in males, it also persisted on Day 2. Starting from Day 3, a constant weight percentage increase was observed in both females and males, but was significantly higher in females.

## 4. Discussion

The study of BW and weight changes during the neonatal period in dogs has received great scientific interest in recent years, highlighting the important influence of the breed on achieved results. The present study, therefore, aimed at investigating and clarifying factors influencing BW and early weight changes in a single breed and in a single breeding facility to avoid the effect of the breed, but also to mitigate the influence of different managements. The same anesthetic protocol and surgery technique, aiming to minimize the mortality at birth, was performed in all cases. Minimizing mortality at birth was also assured by the involvement of 2–3 neonatologists in all cases. Although the authors are not aware of guidelines about the number of staff per puppy during the resuscitation, in the authors’ experience (data not published), the optimal ratio of one neonatologist every 2–3 puppies would be optimal, even if not always applicable. Because of the generally high number of puppies in the enrolled bitches, in all cases, at least 2–3 neonatologists were in charge of newborn assistance. The small number of stillborn observed in the present study seems to support the hypothesis that a well-planned and performed elective cesarean section, coupled with good newborn assistance, could help in reducing puppy mortality at birth, together with implementing selection programs of bitches that could, in the future, be less prone to suffering from dystocia.

The results of the present study confirmed the importance of BW as an early prognostic factor for survival in newborn dogs, in agreement with previous studies reporting BW as an early, highly recognizable prognostic parameter on newborn survival [3,9,10]. Through the ROC curve, the optimal BW cut-off for survival 15 days after birth was identified (*p* < 0.001), with high sensitivity and positive predictive value (PPV), indicating that puppies of this breed with a BW below 340 g were at high risk of death within 15 days after birth. This value is very similar to the 330 g BW reported for the first quartile in Dobermann puppies by [11]. Moreover, it also agrees with the Dobermann breeders’ belief that newborn puppies born with a BW lower than 300 g have a poor survival prognosis. It is known that underweight puppies are more prone to hypothermia, dehydration and hypoglycemia, in turn being more susceptible to infectious diseases [31]. Moreover, the BW being a reflection of the intrauterine life, it is possible that it reflects a non-optimal physical and functional condition of the newborn. The present results indicate that in the population of Dobermann investigated in the present study, puppies with a BW below 340 g should be considered underweight.

The sex of the newborn significantly influenced BW, with heavier males than females, in agreement with data from previous studies reporting males as generally heavier than females in Labradors [14], Great Danes [15], Boxers [13], different breeds [11], and in four large breeds [16]. However, other studies found no differences [9,12], and it was suggested that any sexual dimorphism must be considered as breed-specific [15,17].

However, BW was also significantly (*p* < 0.05) influenced by some maternal parameters, such as age, with older bitches giving birth to heavier puppies. The study by Groppetti et al. [9] showed that heavier puppies were born by bitches aged 2–8 years compared with younger or older ones. Unfortunately, a specific stratification for maternal age was not performed in the present study, so the comparison of results is difficult. However, most of the Dobermann bitches enrolled in the present study were aged between 2 and 8 years, none was younger than 2 years, and only one was 9 years old. Therefore, also inside the range of 2–8 years of age, an influence of increasing age on BW was found, even if a linear conclusion is difficult to depict, given the age distribution of the bitches enrolled. Maternal pre-pregnancy bodyweight also influenced BW, with heavier puppies born by heavier mothers. This result is interesting because all the bitches had pre-pregnancy bodyweights falling within 30–36 kg, according to breed standards. Therefore, even when bitches belonging to the same breed display maternal bodyweight variation, a significant influence on puppies’ BW must be expected. The results of the present study agree with data previously reported [9,10,11,14,15,20]. When the ratio between the mean BW and mean maternal bodyweight, expressed as a percentage, in puppies alive and in puppies dead within 15 days after birth was considered, the 1.3% observed for puppies alive at 15 days after birth was very similar to the 1.4% previously reported for large-body-sized puppies surviving the first week after birth [17], and also, the value observed in puppies dead within 15 days after birth was similar to the one reported by the same author for puppies dead within the first week after birth (1% vs 1.1%, respectively).

Nonetheless, BW was also influenced by litter size, with heavier puppies belonging to smaller litters, in agreement with previous studies in different breeds [9,10,11,12,14,17]. In the present study, litter sizes ranged between five and 15 puppies, so it is reasonable to suggest that larger litters are characterized by lighter puppies, and that in large breeds, the litter size could depend on the prolificacy of the bitch [12]. A significant influence was also observed for the “litter effect”, evidencing that BW may be influenced by each mother, as previously reported [5].

When weight changes are considered, an important result concerns the information about the weight nadir. In all the born alive puppies, the nadir was recorded at about Day 1 after birth, with little differences between females and males. At the nadir, an average of 3.7% of weight was lost in comparison with BW in all puppies, with more pronounced loss in males than females (4.3% vs. 3.4%, respectively), reflecting the typical weight loss reported for the first 24–48 h after birth by most authors [13], and not observed by Schrank and coauthors [12]. Other than this, in the previous literature on human babies, females were reported to gain less weight when compared with males in the first few days after birth, but an underlying cause was not found; meanwhile, another study reported that in human newborns who lost more weight, higher levels of salivary cortisol were detected [32,33]. The weight loss was defined as negatively impacting survival if more than 4% [5,13] and at 5% [4,13], but never exceeding 10% [13]. The difference between sexes seems to suggest that males, although born heavier than females, lose slightly more weight after birth, and the regain of BW is one day delayed in comparison with females. Therefore, the monitoring of newborn puppies in the first days after birth should consider this sex-related difference in these pivotal days after birth.

However, the D1W resulted in significant differences between the two sexes, with males being heavier than females. Because D1W was also significantly associated with survival at 15 days after birth (*p* < 0.001), a ROC curve and the best cut-off value were also defined for the first day after birth. For D1W also, the 340 g cut-off showed the best sensitivity and PPV for death within 15 days after birth. This is an interesting result from a practical standpoint, suggesting that not only the percentage of weight loss may be prognostic for survival, but also that puppies are at risk for death even if they lose weight below the cut-off value. On Day 1 after birth, glucose concentrations were reported to be directly influenced by the weight of the puppies, in turn, increasing the probability of early neonatal death in case of insufficient weight [5]. It can be speculated that Day 1 represents a key functional checkpoint, as it is characterized by a possible decrease in weight (compared with birth) and mirrors the early functional adaptation of the newborn, refining its possibility to survive. Interestingly, similarly to BW, D1W was also influenced by maternal age, maternal bodyweight, litter size [34], and “litter effect” [5]. It could be, then, hypothesized that, on the first day after birth, adaptive processes from intrauterine to extrauterine life are still influenced by maternal characteristics.

Weight changes in dogs are usually reported using weight charts. In the present study, early weight charts were reported for all the born-alive puppies, and separately for females and males, with the latter being slightly lighter than females on Day 3 and Day 6 after birth, with only slight differences between the two sexes from Day 9 onwards. To provide more practical information, the weight changes at certain times were reported, both as actual weight and as a percentage of variation in comparison with BW. This allowed the definition of the time to regain BW and the time required for doubling BW. BW was regained on Day 3 in both females and males. This means that focused attention should be paid to puppies’ weight changes, especially during the first 5 days after birth, to promptly detect those puppies losing too much weight or showing delayed BW recovery [5,17,18]. Bigliardi et al. [13] reported total recovery of BW at 3–5 days after birth in Boxers, followed by a constant increase in weight. For these reasons, in the present study, special attention was paid to the percentage of weight variation during the first 5 days of age. The results showed that, in comparison with BW, weight changes were significantly different across all 5 days (*p* < 0.05), except between Day 1 and Day 2. Therefore, after the significant decrease in weight observed on Day 1, a significant and constant increase followed from Day 3 to Day 5 after birth. This means that, from a practical standpoint, both weight loss and subsequent weight gain should occur rapidly in the first 5 days after birth. Moreover, a significantly higher percentage of weight increase was detected in females than in males from Day 3 to Day 5 (Day 3: *p* < 0.05; Day 4: *p* < 0.01; Day 5: *p* < 0.01). This finding agrees with data previously reported in Bernese Mountain Dogs, with females gaining more weight than males [12].

Finally, the time required for doubling BW occurred on average between Day 12.6 and Day 14, with a slight delay in males compared with females. In Bernese Mountain Dogs, [12] reported weight doubling occurred on average 10 days after birth, with males requiring a longer time compared with females. Therefore, in the population of Dobermann puppies enrolled in the present study, the general perception that puppies should double their BW by around 10 days after birth should be reconsidered. Instead, the findings of the present study agree with results reported by Bigliardi et al. [13] in Boxers, with BW doubling recorded on average 13 days after birth.

## 5. Conclusions

In conclusion, the results from the present study confirmed the important prognostic role of BW, a simple and reliable indicator for newborn puppies’ survival until 15 days after birth, but also that the nadir of weight may have a prognostic role for early survival. Both BW and D1W differ between females and males, as well as the weight changes during the first 3–5 days after birth, and must be considered for the correct monitoring of growth in newborn puppies of this breed. Focusing attention on the weight variation during the first 5 days after birth is important for monitoring the time for BW recovery and the beginning of weight gain. Nonetheless, BW and D1W are influenced by maternal age, maternal pre-pregnancy bodyweight, litter size, and “litter effect”, all factors that, also within a single breed, play a role in early neonatal puppies’ growth.

## Figures and Tables

**Table 1:** Distribution in number and (%) of the 25 deaths that occurred within the first 15 days after birth.

Days After Birth	Number (%) of Deaths
1–3	10 (40)
4–6	7 (28)
7–9	4 (16)
10–12	3 (12)
13–15	1 (4)

**Table 2:** Data, expressed as means ± SDs and (min–max) of maternal age, parity, pre-pregnancy bodyweight, litter size, and the male/female ratio of the 38 bitches.

Maternal Age (Years)	Parity (*n*)	Pre-Pregnancy Bodyweight (kg)	Litter Size (*n*)	M/F
4.5 ± 1.77 (2–9)	2.2 ± 1.15 (1–5)	33.6 ± 2.13 (30–36)	10.6 ± 2.20 (5–15)	1.13

**Table 3:** Data (value, lower and upper 95% CI) from the Youden test to detect the BW cut-off value (340 g) for survival at 15 days after birth.

	BW Cut-Off (340 g)		
	Value (%)	Lower Limit (%)	Upper Limit (%)
Sensitivity	93.3	90.1	95.7
Specificity	75	53.3	90.2
Positive predictive value	98.2	95.3	98.8
Negative predictive value	43.9	33.9	70.7

**Table 4:** Mean changes in weight, expressed in g (95% C.I.), and % of variation, every 3 days in comparison with BW (100%), in the total number of born alive puppies, and in females and males.

	Mean Weight, Expressed in g (95% CI), and in % of Variation in Comparison with BW (100%)		
	All Puppies (*n* = 364)	Females (*n* = 175)	Males (*n* = 189)
BW	428.1 (414.3–442.0) 100%	416.5 (394.9–438.1) 100%	438.8 (421.1–456.5) 100%
Day 1	412.4 (402.9–421.9) 96.3	403.3 (388.9–417.7) 96.6	420.2 (408.0–432.4) 95.7
Day 3	451.4 (443.3–459.5) 105.4	450.8 (438.1–463.5) 108.1	452.1 (444.8–462.4) 103
Day 6	603.9 (596.5–611.3) 141.1	604.0 (592.2–615.8) 144.8	603.8 (595.5–613.1) 137.6
Day 9	721.3 (713.0–729.6) 168.5	718.9 (705.7–732.1) 172.4	723.4 (712.9–733.9) 164.7
Day 12	805.3 (796.2–814.4) 188.1	806.7 (792.6–820.7) 193.5	804.0 (792.2–815.8) 183.1
Day 15	898.8 (879.9–917.7) 210	893.8 (863.8–923.9) 214.4	903.1 (879.3–926.8) 205.7

**Table 5:** Data (value, lower and upper 95% CI) from the Youden test to detect the D1W cut-off value (340 g) for survival at 15 days after birth.

	D1W Cut-Off 340 g		
	Value (%)	Lower Limit (%)	Upper Limit (%)
Sensitivity	90	86.3	93
Specificity	69.6	47.1	86.8
Positive predictive value	97.8	94.4	98.5
Negative predictive value	32	24.8	57.5

**Table 6:** Differences between female and male born-alive puppies in weight percentage variation during the first 5 days after birth.

Days After Birth	Sex of the Puppy	Weight Var %	Sex of the Puppy	Weight Var %	*p*-Value
1	F	−3.4 ± 4.88	M	−4.3 ± 5.96	ns
2	F	0.44 ± 8.72	M	−3.54 ± 8.65	ns
3	F	4.2 ± 10.86	M	1.74 ± 10.9	<0.05
4	F	12 ± 10.81	M	8 ± 11.39	<0.01
5	F	17.9 ± 10.54	M	13.1 ± 11.97	<0.01

## Data Availability

The raw data supporting the conclusions of this article will be made available by the authors upon request.
